# Regulation of Gene Expression through Food—Curcumin as a Sirtuin Activity Modulator

**DOI:** 10.3390/plants11131741

**Published:** 2022-06-30

**Authors:** Anca Ungurianu, Anca Zanfirescu, Denisa Margină

**Affiliations:** 1 Department of Biochemistry, Faculty of Pharmacy, Carol Davila University of Medicine and Pharmacy, Traian Vuia, 020956 Bucharest, Romania; anca.ungurianu@umfcd.ro (A.U.); denisa.margina@umfcd.ro (D.M.); 2 Department of Pharmacology and Clinical Pharmacy, Faculty of Pharmacy, Carol Davila University of Medicine and Pharmacy, Traian Vuia, 020956 Bucharest, Romania

**Keywords:** sirtuin, curcumin, natural compounds as gene regulators

## Abstract

The sirtuin family comprises NAD^+^-dependent protein lysine deacylases, mammalian sirtuins being either nuclear (SIRT1, SIRT2, SIRT6, and SIRT7), mitochondrial (SIRT3, SIRT4, and SIRT5) or cytosolic enzymes (SIRT2 and SIRT5). They are able to catalyze direct metabolic reactions, thus regulating several physiological functions, such as energy metabolism, stress response, inflammation, cell survival, DNA repair, tissue regeneration, neuronal signaling, and even circadian rhythms. Based on these data, recent research was focused on finding molecules that could regulate sirtuins’ expression and/or activity, natural compounds being among the most promising in the field. Curcumin (1,7-bis-(4-hydroxy-3-methoxyphenyl)-1,6-heptadiene-3,5-dione) can induce, through SIRT, modulation of cancer cell senescence, improve endothelial cells protection against atherosclerotic factors, enhance muscle regeneration in atrophy models, and act as a pro-longevity factor counteracting the neurotoxicity of amyloid-beta. Although a plethora of protective effects was reported (antioxidant, anti-inflammatory, anticancer, etc.), its therapeutical use is limited due to its bioavailability issues. However, all the reported effects may be explained via the bioactivation theory, which postulates that curcumin’s observed actions are modulated via its metabolites and/or degradation products. The present article is focused on bringing together the literature data correlating the ability of curcumin and its metabolites to modulate SIRT activity and its consequent beneficial effects.

## 1. Introduction

The concept of *healthy ageing* is defined by WHO as “the process of developing and maintaining the functional ability that enables well-being in older age” [1]. This is becoming more and more important, as the percentage of elders is expected to rise markedly in the next decades and ageing-associated diseases will represent an important financial burden. Both clinicians and health scientists are looking for ways to reduce the risk of age-related metabolic disorders through diet or dietary supplements and, along the way, to build a strong body of data as a basis for the next generation of therapies addressing high incidence age-related diseases [1,2].

It is well-known that our diet greatly influences our health. For many centuries now, empirical observations proved that certain natural products have the ability to positively influence the course of some diseases, to prevent their development or hinder their evolution. Researchers have tried and succeeded to a great extent, to gather the scientific data to support these observations, so we can clearly point to the precise mechanisms of nutraceuticals’ beneficial effects in different pathologies. For example, fruits and vegetables, which are rich in polyphenols, have antioxidant, anti-inflammatory effects, and induce a positive response in the evolution of cardiovascular disease, diabetes, obesity, and numerous other diseases; now, we understand, for the most part, and continue to study the ways through which natural compounds (e.g., curcumin, resveratrol, quercetin, epigallocatechin, etc.) modulate cellular pathways facilitating their better use in preventing/treating diseases with great prevalence nowadays (neurodegenerative disease, metabolic disease, malignancies) [3,4,5,6].

One such very important pathway, which is more and more discussed lately, is the epigenetic regulation, namely the ability of natural compounds to influence enzymes involved in histone structural changes—sirtuins being paramount in this group [7].

Data regarding the mechanisms through which nutrients and dietary patterns may modulate the epigenome are scarce in literature [8,9]. Studying the nutritional factors that have a direct impact on enzymes involved in epigenetic regulation is of great importance, since it can be considered a stepping-stone in developing a guideline for preventing the development of age-related pathologies and ensuring healthy ageing.

The sirtuin family, named after the silent information regulator T1 (SIRT1), includes nicotinamide adenosine dinucleotide (NAD^+^)-dependent protein lysine deacylases, with an important regulatory role in gene expression, DNA repair, cell cycle, mitochondrial function, biogenesis, metabolism and ageing [10]. These proteins are found in numerous different species, from bacteria to humans. The sirtuin family comprises one or two genes in prokaryotes, four members for *Caenorhabditis elegans* (*C. elegans*), five for *Drosophila melanogaster*, and in mammalian cells, seven such enzymes are identified up to now. The first relevant data concerning these genes were obtained for yeast, for which an extra-copy of SIRT2 gene acted as a genome stabilizer and led to 30% lifespan extension [11,12,13].

The lifespan prolonging actions of sirtuins are conserved in all expressing cells, from yeast to mammals, but the complexity of their function increases with the evolution of the organisms [14]. Experimental studies show that sirtuin overexpression is associated with extended lifespan in yeast, *Drosophila*, and *C. elegans* [15,16,17]; SIRT1 overexpression in the hypothalamus increased lifespan for mice by 16% in females and 9% in males [18]. Moreover, decreased sirtuin levels (especially 1 and 6) are reported in senescent cells (mouse embryonic fibroblasts, lung epithelial cells, human endothelial cells, endothelial cells and macrophages exposed to oxidants), being associated with increased DNA damage [19,20,21].

The reports concerning the metabolic and physio-pathological importance of sirtuins lead to intense research toward finding molecules able to up-regulate or better yet modulate these systems. Among these molecules, polyphenols, and very importantly curcumin, were found to be regulators of sirtuin expression/activity. Thus, it is becoming more and more evident that natural compounds could be useful against the pathological alteration of cellular pathways and processes due to their ability to regulate sirtuin’s activity. The aim of the present review is to establish, based on literature data, the link between curcumin and/or its metabolites and the epigenetic mechanisms that they are able to regulate, thus explaining their effects on cell cycle and metabolism and against age-related pathologies.

## 2. Methods

A survey of literature was performed using PUBMED in order to find the most relevant articles reporting preclinical, in vitro and in vivo, and clinical effects of curcumin and its metabolites on the activity of sirtuin family enzymes. Articles were limited to those published in the English language, focusing on the most recent works between 2010 and 2022 (72,5% of the cited material), but not neglecting any older relevant studies. For cell-based studies, the keywords and MeSH terms used were: “sirtuin”, “epigenetic regulation” AND “curcumin”, “curcumin metabolites” AND “cellular effects”, “pathway”, and “signaling.” For preclinical studies, the keywords and MeSH terms used were: “sirtuin”, “epigenetic regulation” AND “curcumin”, “curcumin metabolites” AND “mice,” “rats”. For clinical trials, the used the keywords and MeSH terms were: “sirtuin”, “epigenetic regulation” AND “curcumin”, “curcumin metabolites” AND “clinical trial” AND “effects”, “disease”. The most relevant papers were selected after eligibility analysis and cross-checking. Additionally, literature was reviewed in order to ascertain the key aspects regarding the characteristics of sirtuins and the intake, bioavailability, and metabolism of curcumin; the keywords and MeSH terms used were: “sirtuin”, “sirtuin family”, “sirtuin enzymes”, OR “curcumin” AND “bioavailability”, “metabolism”, “metabolites” and “derivates”.

## 3. Curcumin Mammalian Metabolism

**Figure 1:**
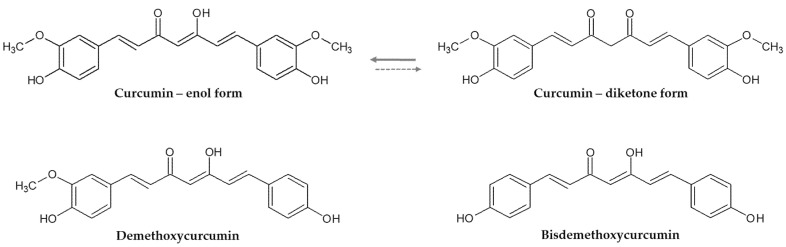
Chemical structure of curcumin and its analogues.

Curcumin—1,7-bis(4-hydroxy-3-methoxyphenyl)-1,6-heptadiene-3,5-dione(diferuloylmethane)—is a natural compound, a diarylheptanoid, belonging to the group of curcuminoids, isolated from the *Curcuma longa* root, proven to have anti-ageing, antioxidant, anti-inflammatory, antihypertensive, anti-diabetes, anti-obesity, antiapoptotic and anticarcinogenic properties [22,23,24]. Curcumin is obtained as an extract from *Curcuma longa* (turmeric) and is used as a food supplement, but also as a condiment or coloring agent in foods and textiles in different parts of the world. Curcumin represents 60–75% of the turmeric extract, the rest corresponding to its analogues—demethoxycurcumin and bisdemethoxycurcumin [25,26,27,28] (Figure 1). In India, curcumin is habitually used, with an average of 100 mg/day and research reports show it to be safe up to 8 g/day [29,30,31].

The structure of curcumin allows for keto-enol tautomerism, as shown in Figure 1, both forms are found as a mixture [27].

Curcumin, as well as its analogues, have a very low water solubility and, consequently, a very low bioavailability, characteristic to most polyphenols, with a significant inter-individual variability. Some studies state that about 1% of the administered doses are found in blood, with peak levels of 3.6 μM obtained after a very high consumption (8 g/day for 3 months) [31,32]; others state that 2 g of curcumin generate a blood Cmax value of 6 ng/mL, and doses of 10–12 g lead to a 50 ng/mL blood concentration [33]. In an effort to overcome the shortcomings of curcumin when it comes to pharmaceutical formulations (low water solubility, poor chemical stability, etc.), modern drug delivery systems were employed. Encapsulation of curcumin in lipid-based nanosystems such as micelles, liposomes, nanoemulsions/complexes, hydrogels or chitosan-based nanocarriers, proved useful in improving its stability and bioavailability [34,35,36].

**Figure 2:**
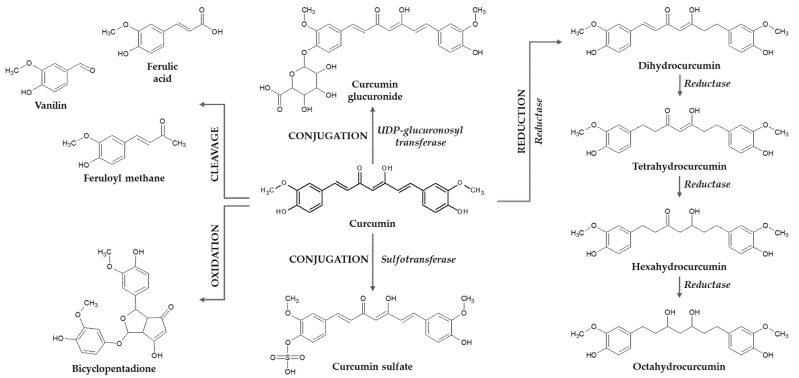
Curcumin metabolism—conjugation, reduction and cleavage.

Curcumin metabolism after oral administration in rats, mice and humans follows two stages (Figure 2)—reduction, as part of phase I metabolism, and conjugation, as a part of phase II metabolism; these processes lead to glucuronide and sulfate conjugates, together with small quantities of octahydrocurcumin, hexahydrocurcumin, tetrahydrocurcumin and dihydrocurcumin [37,38,39,40,41]. The reduced forms of curcumin, generated by alcohol dehydrogenases are either found free or conjugated in the blood [42]. Glucurono-conjugation takes place both in the liver and in the intestinal cells, both in humans and rodents [38]; also, the reductive metabolism can take place under the action of NADPH reductases of the intestinal microbiota, which generates mostly di- and tetrahydrocurcumin [43,44]. Animal studies using radio-labeled curcumin revealed that most of the compound is eliminated through feces and only a small amount by the kidney [45,46].

Taking into consideration the high quantities of curcumin found in the gastro-intestinal tract after administration, researchers postulated that this compound might exert direct modulatory effects of the microbiota and interfere with the gut–brain axis. Mutual two-way interactions between curcumin and the microbiota were reported. Curcumin was found to directly regulate the gut microbiota and its capacity to metabolize the parent compound and generate metabolites—key aspects for curcumin’s pharmacological activity [47,48,49].

Moreover, curcumin is prone to degradation, generating ferulic acid, feruloyl methane, and vanillin, but also autooxidation products, such as bicyclopentadione (Figure 2) [50,51,52,53].

Therefore, most of the curcumin is found in the bloodstream either in the form of metabolites, conjugates or as degradation products, which seem to be responsible for some of the biological effects of this natural compound. Patients with colorectal cancer who received daily oral doses of 3.6 g of curcumin for 4 months presented blood levels of 16 nM glucuronide and 9 nM curcumin sulfate, respectively; for these patients, the urinary concentrations were 1 nM for curcumin, 500 nM for the glucuronide and 40 nM for the sulfate derivative [54].

Studies show that ferulic acid and vanillin yield antioxidant and anti-inflammatory effects but have lower antiproliferative actions compared to the parent compound [55,56,57,58,59]. The curcumin glucuronide is shown to stimulate the expression of antioxidant genes Nrf2, HO-1, and NQO1, but also those involved in epigenetic changes (histone deacetylases–HDAC1, HDAC2, HDAC3, and HDAC4) [60]. The reductive forms have either anti-oxidant properties, through Nrf2 stimulation, or anti-inflammatory, through modulation of COX2, decrease of interleukin release or of Ikβ-α, NF-κβ, and PI3k/Akt/mTOR pathways. They also act on SIRT1 and 2 as well as on HDAC1, thus influencing the epigenetic phenomena associated with curcumin intake [41].

Still, literature shows inferior results when comparing the effects induced by curcumin metabolites, compared to the parent compound on most of the cellular pathways in experimental models. For example, curcumin inhibited TNF-induced NF-κB activation, while its glucuronides had no effects on NF-κB suppression; curcumin, but not the glucuronides, showed anti-proliferative effects on several cancer cell lines [61,62]. Another aspect on curcumin and its metabolites was reported by Jamil et al. [63], who proved in cancer cell lines that curcumin is mainly metabolized through sulfation, and the sulfate exits the cell, thus reducing the intracellular concentration of curcumin and consequently its growth inhibitory effects [41].

## 4. The Sirtuin Family

Sirtuins perform lysine deacetylation of several substrates, together with NAD^+^ hydrolysis (NAD^+^ acting as a co-substrate), thus releasing nicotinamide, which in turn modulates SIRT activity; it is clear that NAD^+^, NADH (generated via mitochondrial processes) and nicotinamide influence the activity of sirtuins [8,64]; for example, upregulation of NAD^+^ biosynthesis significantly prolongs yeast lifespan [65]. The interdependence of sirtuin activity with NAD^+^/NADH ratio suggests the fact that sirtuins can be considered energy sensors [66].

Mammalian sirtuins are located either in the nucleus (SIRT1, SIRT6, and SIRT7), mitochondria (SIRT3, SIRT4, and SIRT5) or cytoplasm (SIRT2), and are catalysts for different metabolic reactions (different “deacylations” of long-chain fatty acids—demalonylation, desuccinylation, decrotonylation, depropyonylation, delipoamidation, or mono-ADP-ribosylation), thus regulating several physiological functions, such as energy metabolism, stress response, cell survival, DNA repair, tissue regeneration, inflammation, neuronal signaling, and even circadian rhythms [14,19,67,68]. Interestingly, some of the sirtuins are able to shuttle from one cell compartment to another; for example, SIRT1 can be found not only in the nucleus, but also in the cytoplasm, while mitochondrial sirtuins can migrate to the nucleus; SIRT3 can migrate from mitochondria to the nucleus as a response to stress (DNA damage) [14,69,70].

The nuclear sirtuins (SIRT1, SIRT6 and SIRT7) are transcriptional regulators that stabilize the chromatin structure [71]; they remove the acetyl fragment from histones as well as non-histone proteins such as transcriptions regulators (NF-κB, MMP-9, FOXO3a and p53) and DNA-repair proteins [67,68]. SIRT1 and 6 modulate the activity of telomere-reverse transcriptase, with a paramount role in maintaining telomere length and deacetylate histone 3 lysine 9 (H3K9) and H3K56 which are required for telomere integrity [19,72,73].

Sirtuins contribute to an improved stress response due to activation of negative regulators of proapoptotic factors (such as p53 and FOXO); for example, SIRT1 deacetylates p53, thus diminishing its DNA affinity, preventing its effect to induce apoptosis and DNA instability. Additionally, the same sirtuin deacetylates FOXO and therefore enhances its ability to act as transcription activator for antioxidant enzymes (SOD) and glutathione peroxidase (GPx) [10,74,75,76]; by activating FOXO, SIRT1 counteracts oxidative stress and promotes DNA repair mechanisms [24,77]. SIRT1 and 6 promote DNA repair by deacetylating repair enzymes, such as poly (ADP-ribose) polymerase (PARP)-1 [73,78,79].

The protective effect against oxidative stress exerted by SIRT1 is also based on its ability to activate through deacetylation gene transcription of PGC-1α (peroxisome proliferator-activated receptor-coactivator 1α), PPAR (peroxisome proliferator-activated receptor), nuclear respiratory factor (NRF), and mitochondrial transcription factor A (TFAM), all of these being involved in the mitochondrial function, as well as in glucose and lipid metabolism [77,80]. Through the modulation of PGC-1α deacetylation, SIRT1 prevents mitochondrial dysfunction and, subsequently, apoptosis [81].

The cytoplasmic and mitochondrial sirtuins perform the same type of deacetylation for enzymatic systems, such as eNOS and AMPK, thus contributing to the regulation of metabolic processes and adaptive cellular responses (cellular anti-stress response, energy metabolism and tumorigenesis) [67,68]. Sirtuins contribute to a reduction of fat accumulation and favor insulin secretion and release through the activation of PPARγ coactivator-1α [10,82]; SIRT4 also regulates mitochondrial glutamine metabolism [83].

**Figure 3:**
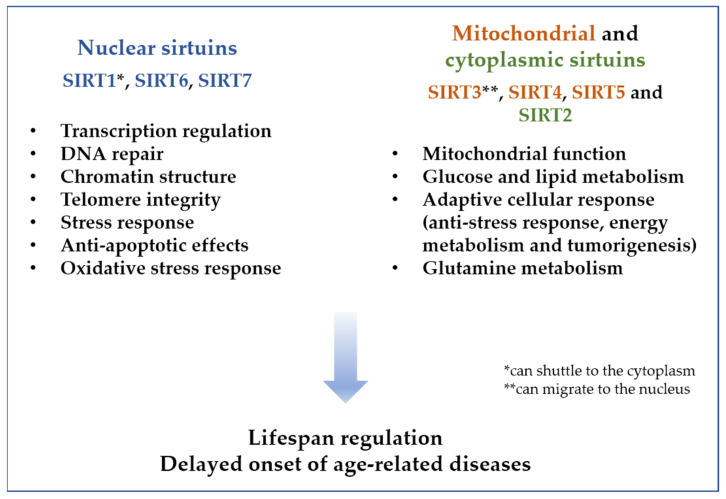
Processes regulated by sirtuins.

The general effect of the processes influenced by sirtuins (Figure 3) is a delayed onset of age-related diseases with the subsequent extension of healthy lifespan, due to their protective effects against age-related pathologies (cancer, liver steatosis, neurodegeneration, cardiovascular disease, diabetes, etc.) [67,68,84]. Therefore, one of the most discussed functions of the sirtuin family is their ability to regulate lifespan; for example, absence of SIRT1 and SIRT6 is correlated with the expression of senescence associated genes and premature ageing [78,85], while SIRT1 and 6 overexpression postponed the senescence for stem cells exposed to stress factors (oxidative stress), but also for human umbilical cord fibroblasts, angiotensin II-treated human coronary artery endothelial cells, primary porcine aortic endothelial cells, stress-exposed lung cells, or bone marrow-derived mesenchymal stem cells and hindered the ageing phenomena for mouse embryonic fibroblasts, human diploid fibroblasts or human endothelial cells [21,72,86,87,88,89,90,91,92,93]. SIRT1 has a higher activity in embryonic stem cells, which decreases in differentiated cells [20]; SIRT3 is also highly expressed in hematopoietic stem cells [94]. Sirtuins 1, 6 and 7 are up-regulated in correlation with calorie restriction; also, their deletion prevents the health benefits of physical exercise and calorie restriction [84,95,96,97,98,99,100].

The impairment of sirtuin activity is correlated with age-related pathological processes, such as cardio-vascular disease, neurodegeneration or cancer [69,101,102]. SIRT1 is the most studied member of this enzymatic family, its dysregulation being involved in inflammation, redox stress, neurodegeneration, fat accumulation, diabetes, tumorigenesis, etc. [10,69]. The SIRT1 level is reduced in liver cells during the ageing process (which is associated with an increase in DNA damage), probably as a result of decreased NAD^+^ availability [103]; also, SIRT1 decreases in ageing arteries and endothelial cells suggesting its involvement in age-related cardiovascular disease [14,104].

If, in most cases, the up-regulation of sirtuins has beneficial effects on cell metabolism and general physiological processes, there are some pathologies where the inhibition of these enzymes rather than their activation might provide beneficial effects; for example, there are studies arguing that SIRT2 inhibition (as provided by AGK2 or MIND4) results in protective actions concerning neurodegenerative diseases, such as Parkinson’s or Huntington’s disease [105,106,107].

## 5. Cellular Effect of Curcumin

According to experimental studies, curcumin is a modulator for the action of different signaling molecules, such as protein kinase C, JNK and advanced glycation end-product (AGE) pathways; it can also act against oxidative stress induced by NADPH oxidase, inhibiting apoptosis via JNK, PI3K-Akt, etc., including in experimental settings associated with high glucose levels [68,108,109]. In a similar manner, curcumin is a direct protector of cardiomyocytes, preventing the oxidative stress-related effects developed under hyperglycemic conditions, thus preventing complications associated with metabolic imbalance in diabetes of metabolic syndrome [24].

Curcumin exerts its antioxidant effects through activation of Nrf2, kelch-like ECH-associated protein 1 (Keap1), a cysteine rich metalloprotein, and antioxidant response element (ARE) [110,111]. Under physiological conditions, Keap1 keeps Nrf2 in the cytoplasm, where its ubiquitination and proteasomal hydrolysis is favored; under the effect of different stressors, Keap1 is blocked through a phosphorylation stage and, consequently, Nrf2 is translocated to the nucleus where it promotes the transcription of genes coding antioxidant molecules (SOD, GSH and GPx and glutathione transferases, HO-1, NADPH, and NAD(P)H dehydrogenase (quinone) 1). Curcumin activates the Keap1/Nrf2 system, through binding of thiol groups from the Keap1 structure, which permits Nrf2 to move to the nucleus and act as a transcription factor for the above-mentioned antioxidant and detoxifying systems [112,113].

Curcumin is also able to reduce the differentiation of preadipocytes, thus acting as an anti-obesity molecule; curcumin decreases the expression of FABP4 and C/EBPα (biomarkers for cell differentiation) in adipocytes [114,115,116], fat accumulation through a decrease of mRNA of the membrane fatty acid transporter [117,118], the expression of fatty acid synthase and glycerol-3-phosphate acyltransferase-1, important enzymes in the synthesis of fatty acids and triglycerides [114,118], these effects being observed for 5, 10 and 20 μM of curcumin. Curcumin, in the same concentrations mentioned before, not only decreases the biosynthesis, but also increases the catabolism of lipids in adipocytes, by increasing the carnitine palmitoyltransferase 1 (CPT-1) expression involved in β-oxidation [115]. Moreover, 0.1 to 10 μM curcumin reduces the NF-κB activity and its nuclear translocation, and so inhibits the recruitment of macrophages into the enlarged adipose tissue and reduces the release of IL-6, TNF-α, MCP-1 from adipocytes [117,119]; the results of in vitro studies are confirmed in animal models of obesity [30].

## 6. Curcumin–Sirtuin Modulatory Effects

### 6.1. Anti-Proliferative and Antiaging Effects

The ability of curcumin to modulate the activity of sirtuins and to facilitate adaptation to certain cellular settings, including stress factors, allows it to be included into the hormetin category [32,120,121]. There are studies focused on the adaptative dose-dependent biphasic effects of curcumin on skin fibroblasts [122]. Additionally, experimental research using a replicative senescence model, as well as a model for premature senescence using doxorubicin, showed that curcumin at low levels of exposure (0.1–1 μM) did not delay senescence and up-regulated sirtuins in vascular smooth muscle cells (VSMCs) and endothelial cells [32]; on the other hand, higher curcumin concentrations (2.5–10 μM) induced senescence in cancer and endothelial cells [123]. Therefore, it can be concluded that, at lower concentrations, curcumin has beneficial, protective, anti-senescence actions, while at higher concentrations it can be pro-senescent and even cytotoxic. Studies showed that endothelial cells are more susceptible to these effects compared to VSMCs, probably due to a cell-specific ability to up-regulate sirtuin levels [32,123,124]. There are also studies demonstrating that the anti-proliferative effects induced by curcumin in VSMCs, due to SIRT7 downregulation, could be a useful approach for mitigating cardiovascular pathologies [125].

Sirtuin up-regulation contributes to the anti-senescence effect reported for curcumin in different experimental studies; for example, curcumin improved keratinocytes differentiation ability in a senescence model [126,127,128]. The lifespan of *C. elegans* was prolonged by curcumin, this effect being abolished by mutations of SIRT2 gene [129].

### 6.2. Anti-Inflammatory and Antioxidant Effects

SIRT1 controls the transcription and consequent expression of inflammatory markers (IL-1, TNF-α, IL-8, IL-6, etc.) by influencing the acetylation of NF-κB and p65 [24,77,80,130]; it also interacts with JAK/STAT pathway, suppressor of cytokine signaling (SOCS), and TLR4/MyD88/NF-κB axis thus modulating the inflammatory networks [112]. Curcumin is one of the molecules able to inhibit STAT3 phosphorylation and thus inhibits the inflammatory pathway; the natural compound acts both in vitro and in vivo to support SOCS1 and 3 expression (as antagonists of JAK/STAT pathway) and through this mechanism, it blocks the release of TNF-α, IL-6, and PGE2 [69,81,112,131,132].

The anti-inflammatory action of curcumin is associated with beneficial effects in different inflammatory pathologies. Administering this natural compound in a mouse model of acute lung injury had a beneficial outcome associated with a decreased expression of NF-κB together with an increased level of SIRT1 [133]; in rat models of hemorrhage shock after lung injury or COPD, curcumin increased pulmonary barrier function, reduced pulmonary oxidative stress and lung inflammation, alleviated COPD by promoting autophagy and inhibiting ER stress and induced the activity of SIRT1 [134,135]. Additionally, in another rat model of COPD (cigarette smoke exposure combined with intratracheal administration of lipopolysaccharide), curcumin induced a reduction of oxidative stress and inflammation markers and increased the expression of SIRT3 [136]. Moreover, curcumin proved protective effects in a rat model of aluminum phosphide induced lung injury, modulated by a decrease in oxidative stress and an increased expression of SIRT1, FOXO1 and FOXO3 [137]. The protective effects were also confirmed in lymphocytes isolated from COPD patients, exposed to 1 µM curcumin, which increased SIRT1 expression and decreased steroid resistance, IFNγ and TNF-α production [138].

Interestingly, besides its anti-inflammatory role, curcumin also acts by stimulating SIRT1 in tert-butyl hydroperoxide-treated rat chondrocytes and consequently inhibits ER stress, thus contributing to an improvement of osteoarthritis development in animal models [139]. 

By stimulating the level of phosphorylated mTOR and stimulating SIRT1 expression in the colon tissue, curcumin had protective effects in a dextran sulfate sodium-induced ulcerative colitis in mice, resulting in a reduction of body weight loss and of disease severity [140]. Additionally, in a model of necrotizing microscopic colitis in newborn rats, curcumin activated the SIRT1/Nrf2 pathway and reduced the TLR4 expression, improving disease evolution [141].

The antioxidant effect of curcumin is also correlated with its ability to modulate sirtuin expression. In a rat model of cisplatin-induced renal impairment, curcumin stimulated SIRT1, SIRT3 and SIRT4 activities [142] reduced oxidative stress and protected the kidney from pathological changes [143]. Additionally, curcumin improved the testis oxidative stress and inflammation induced by cyclosporine use in a rat model of male infertility, by stimulating SIRT1 expression [144]. In a mouse model of sepsis-induced acute kidney injury, tetrahydrocurcumin increased the survival rate, improved the kidney function and ameliorated the renal histological damage, reduced the inflammatory response (IL-1β, IL-6, and TNF-α), alleviated the oxidative stress (measured by MDA level, SOD, GSH, CAT, and GPx activities), and prevented cell apoptosis in renal tissues of septic mice through an increased expression of SIRT1, associated with reduced downstream molecules Ac-p65 and Ac-foxo1 [145]. Moreover, curcumin exerted protective kidney effects in a rat model of gentamicin-induced acute kidney injury, reduced the apoptosis of tubular cells, reduced oxidative stress and induced SIRT1 and Nrf2/HO-1 expression [146].

Curcumin use in mouse and cell models of iron-overload, resulted in an increased SIRT3 expression, associated with a decreased SOD activity, thus protecting against the oxidative stress, and suppressed iron loading-induced autophagy [147].

Studies performed in a diabetes mouse model showed that curcumin treatment modulates SIRT1 through AMPK, thus contributing to an improved glucose absorption and metabolism [148]. Curcumin acts to protect VSMC by activating AMPK and subsequently stimulating ATP and superoxide synthesis, as well as SIRT1 activity; in models of myocardial infarction/reperfusion injury, curcumin reduced oxidative stress damaging effects on mitochondria and lessened the infarction size through a SIRT1-mediated pathway [149,150]. Experimental studies on cardiomyocytes exposed to hypoxia/reoxygenation cycles pointed out the ability of curcumin to reduce apoptosis and oxidative stress in association with an increased SIRT1 expression [151,152]. Additionally, tetrahydrocurcumin, administered in a streptozotocin diabetes mouse model, improved cardiac function and ameliorated myocardial fibrosis and cardiac hypertrophy, accompanied by reduced reactive oxygen species (ROS) generation and increased SIRT1 expression [153]. Curcumin, administered in a model of coronary artery ligation in adult mice, induced the expression of SIRT1 and exerted an anti-fibrotic general effect [154]; the age-related impairment of endothelial cells and VSMC were also abridged through the SIRT1 pathway and the NF-κB inhibitor, and this type of effect was also revealed in muscle cells [32,154,155,156].

All these effects of curcumin could be extrapolated to different pathologies such as diabetes, ischemia/reperfusion injury or cardiac fibrosis. The antiatherosclerotic effects of curcumin were supported by experimental studies showing that this natural compound decreased foam cell formation and intracellular lipid accumulation, and favored cholesterol efflux in macrophages, through an activation of SIRT6 [157] and through activating AMPK-SIRT1-LXRα signaling [158]. Additionally, in a study using a mouse model of atherosclerosis induced by high-fat diet, long term administration of curcumin (80 weeks) protects against the decrease of SIRT1, and also against the increase in senescent cells and inflammation at vascular level [159].

### 6.3. Neuroprotective Actions

Curcumin is one of the natural compounds that has not only hormetic properties, but also, specifically, neuro-hormetic properties, improving in a dose-dependent manner the adaptive stress response through an increased expression of: antioxidant enzymes, sirtuins, FOXO transcription factors, chaperones, neurotrophic factors and anti-apoptotic proteins and, thus, suppressing the development of pathological processes in animal/cell models of neurodegenerative disorders such as Alzheimer’s and Parkinson’s diseases [160]. Sirtuins, PGC-1α, oxidative stress, inflammation and mitochondrial dysfunction are potential targets and, through their modulation, curcumin could exert neuroprotective effects against the pathological pathways associated with neuronal lesions/neurodegeneration (Alzheimer’s, Parkinson, multiple sclerosis, etc.) [161,162].

Curcumin, together with other natural compounds, may possess the ability to reduce the hallmarks of neuronal damage and slow down the characteristic cognitive decline and positively modulate the pathological pathways in Alzheimer’s (amyloid deposits, neurofibrillary tangles, synaptic loss, inflammation, regulating autophagy and extensive oxidative stress) [4,163], some of these effects are being associated with increased SIRT expression [164]. The antioxidant and other beneficial effects associated with increased SIRT2 level were pointed out in the hippocampus of adult rats treated with curcumin [165].

Some other neuronal protective effects were also observed; for example, curcumin attenuated memory deficits in amyloid-induced neuronal metabolic dysfunction and improved cognition in transgenic mice through increasing SIRT3 activity [166], improved mitochondrial membrane potential and inhibited apoptotic cell death in Aβ25-35 treated neurons, activated the expression of SIRT1 and subsequently decreased the expression of Bax (apoptosis regulator) in the presence of Aβ25-35 [167]. Additionally, curcumin reduced glutamate excitotoxicity in cultured neurons, through an increased expression of SIRT1 and reduced the level of ac-PGC1α due to its deacetylation under the effect of SIRT1 [168]. Curcumin reduced the levels of inflammatory markers and induced SIRT1 and Bax expressions in a cerebral ischemia/reperfusion injury rat model, thus demonstrating clearly neuroprotective effects [169]. Bisdemethoxycurcumin, a curcumin derivative, manifested also neuroprotective effects in experimental models of Alzheimer’s disease, the effects being associated with increased SIRT1 activity and modulation of GSH and SOD [170].

Curcumin’s effects on sirtuins were also pointed out in clinical settings; in a randomized, double-blind clinical study including 72 patients with PCOS, results proved that administering 1500 mg curcumin, 3 times/day significantly reduced oxidative stress markers, increased gene expression for PGC1-α and also a non-significantly increased gene expression of SIRT1 and the activity of the SOD enzyme [171]. Similar results were obtained for animal models; female mice treated i.p. with curcumin showed increased expression for SIRT1 and 3, associated with an increase in number of follicles and oocyte maturation, fertilization and embryo development as well as decreased oxidative stress [172].

### 6.4. Anti-Cancer Potential

Related to the anti-cancer potential of curcumin, there are some studies proving that the natural compound induces apoptosis in cancer cells [156,173]; experimental studies show that SIRT1 is overexpressed in colon cancer cells, compared to normal cells; knockdown of SIRT1 led to the decreased viability and migration of cancer cells as well as a decrease in tumor volume and growth rate. Curcumin treatment reduced the expression of SIRT1 protein in colon cancer cells and facilitated the proteasomal degradation of oncogenic SIRT1 [174].

### 6.5. Other Effects

Curcumin activates the SIRT1 pathways in all cellular compartments; experimental and preclinical studies pointed out that SIRT1, as a cardioprotective factor, is inhibited by stress factors associated with increased risk of myocardial infarction; reduced SIRT1 activity is observed in inflammatory settings, as well as during the ageing process, both phenomena being associated with oxidative stress. Hyperglycemia and hypercholesterolemia inhibit SIRT1 and curcumin is able to restore its activity, thus acting as a protective agent against ischemia-reperfusion injury. On the other hand, SIRT1 contributes to an enhancement of mitochondrial processes and reduces oxidative stress and inflammation in cardiomyocytes; also, curcumin treatment of hyperglycemia/osmotic stress exposed cardiomyocytes exerted a protective effect, improving cell viability and also enhancing SIRT3 expression and activity [175,176].

The reported cellular effects can be correlated with results from animal studies, SIRT1 stimulating insulin secretion, while reducing glucagon secretion, both mechanisms leading to an increased cellular use of glucose and a reduction glycemia [22,24,88,177,178,179,180]. Protective effects regarding mitochondrial metabolism were also reported by a study employing an acute liver injury model in mice, when curcumin reduced oxidative stress and apoptosis through modulation of hepatic SIRT1 activity [181]. Additionally, in a model of nonalcoholic fatty liver disease, curcumin reduced inflammation and steatosis, through an increase in SOD1 and SIRT1 expression [182]; another experiment showed that dietary curcumin reduced hepatic steatosis and improved mitochondrial function via SIRT3 activity, in a hepatic steatosis model in postnatal overfed rats [183]. Additionally, curcumin is one of the phytochemicals able to induce a calorie restriction-type metabolic state, through an induction of AMPK, yielding a tumor suppressing effect. These types of metabolic effects are determined by a SIRT1 induction, associated with the reduction of IL-6 and NF-κB, as well as an increased PPARα level [184].

Some of curcumin’s effects on sirtuin signaling pathways are still under debate, since some of the experimental and preclinical results are not in complete agreement; for example, Yin et al. [185] pointed out that 30-50 μM curcumin did not alter SIRT2 expression in mouse bone marrow-derived macrophages treated with nigericin for NLRP3 inflammasome activation. So, even if other literature data demonstrate that curcumin is a histone deacetylase inhibitor [186], the above mentioned results suggest that inhibition of histone deacetylases did not significantly influence the NLRP3 inflammasome activation [185]. On the other hand, Wang X et al. [187] demonstrated that curcumin promotes cell survival and mitochondrial quality of bone marrow mesenchymal stem cells subjected to hypoxic preconditioning, through the up-regulation of PGC-1α and SIRT3, thus accelerating the wound healing process in mice. Moreover, curcumin was proven to support the metabolic activity and muscle force gain in a mouse model of cancer-induced cachexia; this effect being correlated with an increase in SIRT1 activity [188]; results were similar in a mouse model of disuse muscle atrophy, curcumin stimulating the number of muscle cells and SIRT1 activity [189], and also in a study proving that muscle performance was improved by curcumin through a stimulation of SIRT1 activity [190].

Experimental and preclinical results regarding the effects of curcumin on sirtuin activity, along with experimental models and doses used, are presented in Table 1 and Table 2.

## 7. Conclusions

Recent research established the fact that sirtuins are keynote players in the ageing process, acting in order to delay cellular senescence and extending cellular longevity, while their activity is dysregulated in different pathologies associated with the ageing process. The discovery of more and more details regarding the structure and function of sirtuins, their involvement in the beneficial effects of calorie restriction, as well as their modulation through mitochondrial processes generating NAD^+^/NADH, unraveled the perspective that molecules influencing their action could be used to combat the decline of cellular pathways encountered along the timeline of every living organism. Some of the most abundant and easy to access sirtuin modulators are natural compounds, especially polyphenols, and in this class, curcumin seems to be an important molecule with relevant modulatory action on the sirtuin-mediated cellular processes, with a good perspective to become an important tool to actively protect against age-related diseases. There is still much to be learned about the functions of sirtuins and of the molecules that modulate their activity.

We conclude that curcumin can positively influence numerous physio-pathological processes, contributing to the prevention of cardiovascular, malignant and neurological damage, sirtuins being its key targets in these respects. Future research will be able to tell if curcumin can be used as clinical modulator of sirtuin activity. Furthermore, there is much to be unraveled regarding the potential of curcumin metabolites to modulate the expression and the activity of sirtuins, thus contributing to the overall effects induced in senescent/young cells by the parent compound. Pharmaceutical studies regarding the potential changes in bioavailability through modern drug delivery systems represent an emerging topic that should develop and merge with the ongoing biochemical research regarding curcumin’s effects on cellular signaling pathways. Additionally, the assessment of the pharmacokinetic profile of curcumin and other natural compounds in healthy subjects, but also in the elderly, is critical for ascertaining if the observed effects of these molecules are determined by the parent compound, their metabolites or, to a certain degree, of both.

The in vivo effect of every sirtuin modulator found, including curcumin and its derivatives, the extent to which they can influence the activity of sirtuins, should be assessed through clinical trials. These types of studies are paramount in the search for molecules able to contribute to achieving healthy ageing and improving quality of life, an objective sought by clinicians and researchers alike over the last 20 years.

## Figures and Tables

**Table 1:** Preclinical results regarding the effects induced by curcumin in animal models, through modulation of sirtuin activity and expression.

Animal Model	Curcumin Dosage	Mechanism of Action	Reference
Streptozotocin-induced diabetes in rats	100 mg/kg/day	↑ SIRT1 level ↑ SOD activity and ↓ MDA level ↑ expression of NQO1 and Nrf2 ↓ oxidative stress ↓ cardiomyocyte apoptosis in of type 2 diabetes rats	[24]
Rat model for ischemia/reperfusion injury (IRI) and also testing on isolated cardiomyocytes	In vivo study: curcumin 200 mg/kg p.o., 10 days In vitro study: 0.25–10 μM pretreatment with curcumin	↓ myocardial infarct size cardioprotective effect against IRI due to the activation of SIRT1 signaling ↑ SIRT1, COX, SDH ↓ Bax Effects were abolished by SIRT1 siRNA treatment of cells	[150]
Genetic model of diabetes in mice (db/db)	0.75% curcumin in diet, 8 weeks	↓ NF-κB ↑ AMPK, PPARγ, ↑ glucose metabolism no effects on SIRT1 and PGC-1α in the liver of db/db mice;	[148]
Mouse model of arteriosclerotic disease using aged C57BL/6J mice fed with high fat diet	0.1% curcumin in diet, 80 weeks	↑ HO-1 in the aorta with ↓ of oxidative stress ↑ SIRT1 expression in aorta ↓ inflammation	[159]
Diet induced obesity model using male wild-type C57BL/6J mice (8–10-week-old) Male ob/ob C57BL/6J mice (3–5-week-old).	3% curcumin in diet, 60 days	↓ body fat ↑ insulin sensitivity and glycemic control ↑ SIRT1 expression, HSP70, HSP90, FOXO1α in white adipose tissue ↑ ER stress response ↓ Hepatic NF-κB activity	[191]
Rat osteoarthritis model by anterior cruciate ligament transection	50 mg/kg and 150 mg/kg i.p., 8 weeks	↑ SIRT1 expression ↓ degeneration of articular cartilage ↓ chondrocyte apoptosis	[139]
Mouse model of cancer cachexia	1 mg/kg/day i.p., 15 days	↑ SIRT1 in gastrocnemius and soleus muscles	[188]
Mouse model of disuse muscle atrophy	1 mg/kg/day i.p., 14 days	↑ SIRT1 activity ↑ number of muscle progenitor cells	[189]
Exercise performance and endurance in male Wistar rats	100 mg/kg, 6 weeks followed by intense effort	↑ PGC-1α, thioredoxin-1, SIRT1, Nrf2, ↑ SIRT1 expression ↑ muscle performance	[190]
Iron overload cell and mouse models	200 mg/kg dissolved in corn oil, p.o., 4 weeks	↑ SIRT3 ↓ SOD2	[147]
Cisplatin induced renal impairment in rats	200 mg/kg/day p.o., 3 days	↑ SIRT3 activity ↓ impairments of mitochondrial bioenergetics, ultrastructure, redox balance, dynamic ↑ kidney protection	[143]
Rat model of gentamicin-induced acute kidney injury	100 mg/kg intragastric, 8 days	↑ SIRT1 expression ↑ Nrf2/HO-1 ↓ apoptosis of renal tubular cells improved renal parameters	[146]
Mouse model of sepsis-induced acute kidney injury	120 mg/kg tetrahydrocurcumin i.p., 24 h	↓ IL-1β, IL-6, and TNF-α ↓ MDA level, SOD, GSH, CAT, and GPx activities ↑ SIRT1 expression	[145]
Dextran sulfate sodium-induced ulcerative colitis in mice	50 mg/kg/day in diet, 14 days	↑ phosphorylated mTOR and SIRT1 expression in the colon tissue ↓ body weight loss and attenuated the severity of the disease	[140]
Necrotizing microscopic colitis in newborn rats	20 mg/kg and 50 mg/kg intragastric	↑ activation of the SIRT1/NRF2 pathway, with improved disease evolution ↓ TLR4 expression	[141]
Acute liver injury model in mice	200 mg/kg i.p., 1h after D-galactosamine (D-GalN)/lipopolysaccharide (LPS)-induced acute liver injury	↓ hepatic SIRT1 ↑ SOD activity, ↓ AST level ↑ mitochondrial function ↓ apoptosis	[181]
Hepatic steatosis in postnatal overfed rats	2% curcumin in diet	↓ hepatic steatosis ↑ mitochondrial function through SIRT3	[183]
Diet induced non-alcoholic fatty liver disease (NAFLD) model in mice	100 mg/kg p.o., 3 weeks	↓ severity of hepatic steatosis (through relieving the dependence of O-GlcNAcylation on NF-κB in inflammation signaling) ↓ severity of hepatic inflammation ↑ SOD1 and SIRT1 expression	[182]
Acute lung injury in mouse	100mg/kg/day and 200mg/kg/day p.o., 5 days	↓ NF- κ B ↑ SIRT1	[133]
Rat model of COPD induced by cigarette smoke exposure combined with intratracheal administration of lipopolysaccharide	100 mg/kg p.o., 30 days	↑ mRNA and protein expression of PGC-1α and SIRT3 in the skeletal muscle tissues ↓ oxidative stress (MDA, Mn SOD, SOD, GPx, CAT) ↓ inflammation (IL-6 and TNF-α)	[136]
Rat model of aluminum phosphide induced lung toxicity	100 mg/kg/day p.o. (curcumin and nanocurcumin), 7 days	↑ SIRT1, FOXO1, FOXO3 in lung tissue ↑ antioxidant capacity and antioxidant enzymes (CAT, GPx),	[137]
Acute lung injury followed by hemorrhage shock and resuscitation rat model	50, 200, or 400 mg/kg p.o., 4 days	↑ lung SIRT1 ↑ pulmonary barrier function, ↓ pulmonary oxidative stress and lung inflammation	[134]
Healthy female mice	100 mg/kg/day i.p., for 6, 12 and 33 weeks	↑ ovarian volume and number of follicles ↑ oocyte maturation, fertilization and embryo development ↓ oxidative stress ↑ expression of GDF-9, BMP-15, SIRT1 and SIRT3	[172]
Rat model of male infertility (using cyclosporin)	40 mg/kg p.o., 28 days	↑ SIRT1 ↓ oxidative stress and inflammation in testis	[144]
Rat model of cerebral ischemia/reperfusion injury	50 mg/kg i.p., 5 days	↓ TNF-α, IL-6 ↑ mitochondrial membrane potential, mitochondrial complex I activity, and mitochondrial cytochrome c levels, ↑ SIRT1 and Bcl-2 expression	[169]

**Table 2:** Experimental results regarding the effects induced by curcumin in cell models, through modulation of sirtuin activity and expression.

Cell Model	Curcumin Dosage	Mechanism of Action	Reference
H9c2 cardiomyocytes exposed to high-glucose (25 mmol/L) and high-fat (500 μmol/L saturated free fatty acid palmitate)	2.5–20 μM	↑ SIRT1-FOXO1 and PI3K-Akt pathways that were ↓ by the pathological conditions	[24]
HepG2 cells exposed to high-glucose (10mM and 30mM glucose)	5 μM and 10 μM	↑ Cell viability, ↑ SIRT3, PGC-1a, CREB, GPx1, and SOD2	[175]
Human THP-1 macrophages exposed to oxLDL for foam cell generation	0–80 μM	↑ SIRT6 expression ↑ cholesterol efflux through miR-125a-5p/SIRT6 axis and regulate the expression of ABCA1	[157]
Vascular smooth muscle cells	2.5–10 μM	↑ AMPK, superoxide level, ATP production ↑ SIRT1, NAD^+^ level	[149]
Vascular smooth muscle cells	5 μM	↓ SIRT7 cytostatic effect ↑ DNA methyltransferase 2 (DNMT2)	[125]
Vascular smooth muscle cells and endothelial cells	0.1 and 1 μM	↓ IL-8 and VEGF ↑ phosphorylation of SIRT1 and the level of SIRT6 (after 2-18h), ↑ SIRT3 (1-3 days)	[32]
HUVECs exposed to 100 µM H2O2 for senescence induction	5, 10 and 25 µM, 48 h	↑ SIRT1 expression ↓ oxidative stress	[156]
Rat chondrocytes isolated from the cartilage of rat hip joint	0, 5, 10, 20, 25, and 50 μM/L	↓ apoptosis ↓ ER stress-related biomarkers CHOP, GRP78, and ATF4 ↑ SIRT1 expression	[139]
Rat bone marrow mesenchymal stem cells	10 μM curcumin, 2 h, followed by hypoxic exposure treated cells were used for wound healing	↑ mitochondrial quality via promoting mitochondrial fusion and metabolic activity through PGC-1α and SIRT3 modulation	[187]
Human neuroblastoma cells SK-N-SH exposed to acrolein	5 μM-20 μM	↓ acrolein toxicity ↑ Nrf2, NF-κB, and SIRT1 expression	[192]
Colon cancer cells	1 or 10 μM, 3 h	↓ SIRT1 expression ↓ SIRT1 catalytic activity ↓tumor volume and invasivity	[174]
Cancer cells (FaDu and Cal27)	7 μM, 10 μM, 6, 12, 24 and 48 h	↑ SIRT1 expression ↑ caspase 8 and 9 activity	[156]
Primary culture of rat neurons exposed to glutamate excitotoxicity	10 μM, 20 μM 2h pretreatment	↓ cell death and apoptosis ↑ mitochondrial function ↑ SIRT1 expression ↓ ac-PGC1a	[168]

## Abbreviations

Nrf2 (nuclear factor erythroid 2-related factor 2), HO-1 (heme oxygenase 1), NQO1 (NAD(P)H dehydrogenase (quinone) 1), HDAC (histone deacetylase), Ikβ-α (inhibitor of nuclear factor kappa), COX2 (cyclooxygenase-2), NF-κβ (nuclear factor kappa-light-chain-enhancer of activated B cells), PI3k (phosphoinositide 3-kinase), Akt (previously known as protein-kinase B), mTOR (mammalian target of rapamycin), TNF (tumor necrosis factor), MMP-9 (matrix metallopeptidase 9), FOXO (forkhead family of transcription factors), SOD (superoxide dismutase), PARP (poly (ADP-ribose) polymerase), PGC-1α (peroxisome proliferator-activated receptor-coactivator 1α), PPAR (peroxisome proliferator-activated receptor), TFAM (mitochondrial transcription factor A), NOS (nitric oxide synthase), AMPK (5’ adenosine monophosphate-activated protein kinase), GPx (glutathione peroxidase), JNK (c-Jun N-terminal kinase), AGE (advanced glycation end-product), Keap1 (kelch-like ECH-associated protein 1), ARE (antioxidant response element), GSH (reduced glutathione), FABP4 (fatty acid binding protein 4), C/EBPs (CCAAT enhancer binding protein family), MCP-1 (monocyte chemoattractant protein-1), VSMC (vascular smooth muscle cells), JAK/STAT (Janus kinase/signal transducers and activators of transcription), SOCS (suppressor of cytokine signaling), TLR4 (Toll-like receptor 4), MyD88 (an adapter protein that mediates signal transduction for most TLRs), COPD (chronic obstructive pulmonary disease), ER stress (endoplasmic reticulum stress), IFNγ (interferon gamma), MDA (malondialdehyde), CAT (catalase), Aβ (amyloid beta), Bax (bcl-2-like protein 4), NLRPs (NOD-like receptors pyrin containing domain), IRI (ischaemia/reperfusion injury), DNMT2 (DNA methyltransferase 2), AST (aspartate aminotransferase), GDF-9 (growth differentiation factor 9), BMP-15 (bone morphogenetic protein 15), CHOP (C/EBP homologous protein, also known as DNA damage-inducible gene 153-GADD153), GRP78 (glucose-regulated protein 78 kDa), ATF4 (activating transcription factor 4, tax-responsive enhancer element B67).
